# Enhanced Oral Bioavailability of the Pharmacologically Active Lignin Magnolol via Zr-Based Metal Organic Framework Impregnation

**DOI:** 10.3390/pharmaceutics12050437

**Published:** 2020-05-09

**Authors:** Joshua H. Santos, Mark Tristan J. Quimque, Allan Patrick G. Macabeo, Mary Jho-Anne T. Corpuz, Yun-Ming Wang, Tsai-Te Lu, Chia-Her Lin, Oliver B. Villaflores

**Affiliations:** 1 The Graduate School, University of Santo Tomas, España Blvd., Manila 1015, Philippines; jhsantos120@gmail.com (J.H.S.); mtcorpuz@ust.edu.ph (M.J.-A.T.C.); 2 Phytochemistry Laboratory, Research Center for the Natural and Applied Sciences, University of Santo Tomas, España Blvd., Manila 1015, Philippines; 3 Mindanao State University-Iligan Institute of Technology, Tibanga, Iligan City 9200, Philippines; mtjquimque@gmail.com; 4 Laboratory for Organic Reactivity, Discovery and Synthesis (LORDS), Research Center for the Natural and Applied Sciences, University of Santo Tomas, España Blvd., Manila 1015, Philippines; agmacabeo@ust.edu.ph; 5 Pharmacology Laboratory, Research Center for the Natural and Applied Sciences, University of Santo Tomas, España Blvd., Manila 1015, Philippines; 6 Department of Pharmacy, Faculty of Pharmacy, University of Santo Tomas, España Blvd., Manila 1015, Philippines; 7 Department of Biological Science and Technology, Institute of Molecular Medicine and Bioengineering, Center for Intelligent Drug Systems and Smart Bio-devices (IDS2B), National Chiao Tung University, Hsinchu 30010, Taiwan; ymwang@mail.nctu.edu.tw; 8 Institute of Biomedical Engineering, National Tsing Hua University, Hsinchu 30013, Taiwan; ttlu@mx.nthu.edu.tw; 9 College of Science, Chung Yuan Christian University, Zhongli District, Taoyuan City 320, Taiwan; chiaher@gapps.ntnu.edu.tw; 10 Department of Biochemistry, Faculty of Pharmacy, University of Santo Tomas, España Blvd., Manila 1015, Philippines

**Keywords:** magnolol, metal organic framework, bioavailability, toxicity, Uio-66(Zr)

## Abstract

Bioavailability plays an important role in drug activity in the human body, as certain drug amounts should be present to elicit activity. However, low bioavailability of drugs leads to negligible use for human benefit. In this study, the diversely active neolignan, magnolol, was impregnated onto a Zr-based organometallic framework [Uio-66(Zr)] to increase its low bioavailability (4–5%) and to test its potential acute oral toxicity. Synthesis of Uio-66(Zr) was done through the solvothermal method while simple impregnation at different time points was used to incorporate magnolol. The loading capacity of Uio-66(Zr) at 36 h was found to be significantly higher at 72.16 ± 2.15% magnolol than in other incubation time. Based on the OECD 425 (limit test), toxicity was not observed at 2000 mg kg^−1^ dose of mag@Uio-66(Zr) in female Sprague Dawley rats. The area under the curve (AUC) at 0–720 min of mag@Uio-66(Zr) was significantly higher than the AUC of free magnolol. Moreover, relative bioavailability increased almost two-folds using Uio-66(Zr). Unconjugated magnolol was found in the liver, kidney, and brain of rats in all treatment groups. Collectively, Uio-66(Zr) provided a higher magnolol bioavailability when used as drug carrier. Thus, utilization of Uio-66(Zr) as drug carrier is of importance for maximal use for poorly soluble and lowly bioavailable drugs.

## 1. Introduction

**Figure 1:**
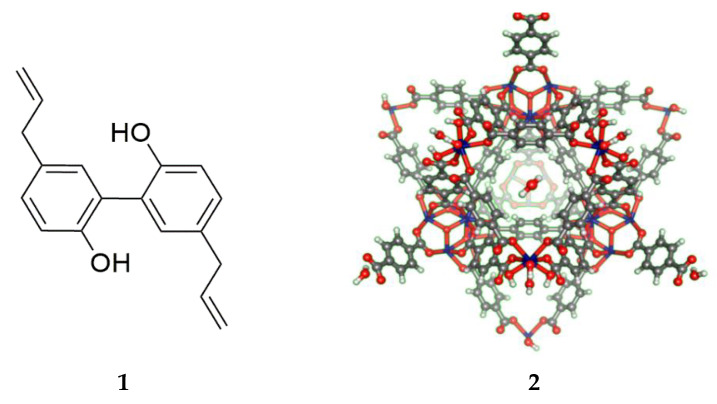
The structure of the (**1**) neolignan, magnolol (4-Allyl-2-(5-allyl-2-hydroxy-phenyl)phenol) and (**2**) Uio-66(Zr). (as visualized using ACD/Chemsketch ver 2018.1, Canada).

Despite the numerous drug compounds available, 60–70% of them are not readily soluble in aqueous media, resulting to a lower absorption rate and bioavailability [1]. Most neuroactive natural products such as magnolol, allium compounds, berberine, curcumin, genistein, ginsenoside K, and resveratrol possess low bioavailability mainly due to their poor solubility in water, low permeation, high first-pass effect and high presystemic excretion [2,3]. Magnolol, shown in Figure 1, exhibits a spectrum of biological and pharmacological activities including smooth muscle relaxation [4], inhibition of fungal and microbial growth [5], suppression of asthmatic attacks [6], interception of reactive oxygen species production [7], inhibition of cancer cell proliferation [8], and halting of inflammatory responses [9]. Similar to other natural products, the major setback in the use of magnolol as a neuroactive drug is its limited bioavailability [10,11]. 

Approaches to increase the bioavailability of magnolol include the use of micellar particles [12,13], mesoporous silica [14], nanoparticles [15,16], solid dispersions [17,18], and liposomes [19]. As part of our continuing efforts to increase the bioavailability of biologically active natural products with limited solubility in aqueous systems, a metal organic framework (MOF) was utilized in this study as drug delivery system for crystalline yellow magnolol (**1**). MOFs are porous coordination networks [20] or coordination polymers [21] composed of a central metal ion and organic linkers. MOFs are characterized to have high porosity, thermal stability, discrete ordered structure, ultra-low density, large internal surface area (above 6000 m^2^ g^−1^), ease of synthesis, and various applications. MOFs also possess high degree of crystallinity and order of units [22]. These characteristics through a potential pocket enable MOFs to facilitate entry and binding of drugs (guest molecules) [23]. In this study, a zirconium-based MOF Uio-66(Zr), was found to have high porosity, shear, and thermal stability [24]. Zirconium and its complexes exhibit low systemic toxicity resulting from its poor water solubility [25,26,27]. Several studies revealed that Uio-66(Zr) (**2**) can be used as a drug carrier system for anticancer [28,29], anti-inflammatory [30], and antibacterial agents [31]. It was also able to enter cells via clathrin-mediated endocytosis [27].

In this paper, we performed a solvothermal method for Uio-66(Zr) synthesis and the simple impregnation technique to incorporate magnolol. Pure Uio-66(Zr) and mag@Uio-66(Zr) were characterized and compared to assess magnolol impregnation by thermogravimetric analysis (TGA), powder X-ray diffraction (PXRD), and nitrogen sorption isothermal techniques. Our study reports for the first time the preparation of a magnolol-loaded metal organic framework based on zirconium with increased oral bioavailability. 

## 2. Materials and Methods

### 2.1. Material and Reagents

Magnolol (≥ 98%) was purchased from Xi’an Lyphar Biotech Co., LTD. (Xi’an, China). Zirconium chloride (ZrCl_4_, 99.9%), and trifluoroacetic acid (TFA, 99%) were purchased from Alfa Aesar, Inc. (Shanghai, China). Terephthalic acid (98%) used in this study was purchased from Tokyo Chemical Industry Co., LTD. (Kumagaya, Saitama, Japan). Dimethylformamide, anhydrous (DMF, 99.8%) was purchased from Merck LTD. (Taipei, Taiwan). Acetonitrile, HPLC grade (99.9%), and hydrochloric acid (HCl, 35.5–38.1%) was purchased from JT Baker (Phillipsburg, NJ, USA). Ethanol (95%) was purchased from Tianjin Jingming Chemical Co., LTD. (Tianjin, China). Ultrapure water used was prepared from with Milli-Q ultrawater system. All reagents were used without further purification.

### 2.2. Animals

Female non-nulliparous Sprague Dawley (SD) rats (300 ± 50 g) and male SD rats (200 ± 50 g) were purchased from Laboratory Animal Facility, Research & Biotechnology Group, St. Luke’s Medical Center (Manila, Philippines). All rats were provided distilled water ad libitum. The animals were housed at controlled temperature of 25 ± 2 °C and relative humidity of 45 ± 5% for 7 days prior to experimentation. All animal experiments were reviewed and approved by the Institutional Animal Care and Use Committee (IACUC) of the University of Santo Tomas (AR-2017-352, dated 21 September 2017). Prior to dosing, animals were fasted for 12 h and were only given distilled water.

### 2.3. Methods

#### 2.3.1. Synthesis of Uio-66(Zr)

UiO-66(Zr) (**2**) was synthesized using the solvothermal method as previously described by Ahmed et al. (2016) [32]. Two millimoles of ZrCl_4_ and 4 mM of terephthalic acid were dissolved in 50 mL of DMF acidified with 2 mL 12M HCl. The resulting solution was transferred to a 100 mL Teflon-lined autoclave and was heated at 180 °C for 24 h. The MOF powder was filtered and washed with DMF (1 g of MOF: 50 mL DMF). The residue was dried at 150 °C for 5 h. The solution was centrifuged at 5000 rpm for 10 min, the supernatant liquid was discarded, and the powder was dried for 12 h in vacuum oven at 150 °C.

Activation was done by washing the MOF with 95% ethanol (10 mg MOF: 1 mL ethanol). The mixture was agitated using a vortex mixer for 30 min. The resulting solution was centrifuged at 10,000 rpm for 5 min to separate the powder and supernatant liquid. The procedure was repeated 5 times and the obtained MOFs were dried at 110 °C vacuum oven for 24 h [33].

#### 2.3.2. Magnolol Impregnation and Quantification

Magnolol (**1**) was loaded into the Uio-66(Zr) framework through simple impregnation. In particular, 1 mg of Uio-66(Zr) was mixed with 100 µL of magnolol solution (20 mg/mL in ethanol) at 75 rpm in a closed tube, resulting in a 1:2 (Uio-66(Zr):magnolol) ratio. The mixture was mixed for 12, 24, 36, and 48 h. The mixture was centrifuged at 10,000 rpm for 5 min to separate the supernatant liquid and MOF. Addition of 200 µL of ethanol was done with careful pipette in-out to remove the magnolol adhered outside the Uio-66(Zr) and the container. The mixture was recentrifuged again to separate the washing and MOF. The washing was combined with the previously collected supernatant liquid and kept for subsequent analysis. The collected MOF was labeled as Mag@Uio-66(Zr). The amount of magnolol loaded into the MOF was determined indirectly by measuring the amount of unentrapped magnolol in the combined washing and supernatant liquid [14,34].

The amount of magnolol impregnated into the MOF was determined by detecting the amount of free magnolol present in the supernatant liquid by using the high performance liquid chromatography method using HPLC Agilent 1100 series with Luna 5 µm C18(20) 100 angstrom, 250 mm × 4.6 mm (H15-216693) equipped with UV–VIS diode array detector (Agilent Technologies, Germany). Acetonitrile and water with 0.1% trifluoroacetic acid (80:20) were used as mobile phases [14,35].

(1) $$\mathrm{Drug}\;\mathrm{Loading}\;\mathrm{Percent}=\frac{TA-SA}{TA}\times 100$$

where *TA*—total amount of magnolol in solution (mg); *SA*—amount of magnolol in supernatant (mg).

#### 2.3.3. Characterization of Magnolol-Loaded MOF

##### Nitrogen Sorption Isotherms

Samples were outgassed under high vacuum at 60 degrees for 48 h to remove the residual solvents attached in the MOF using the Quantachrome Nova 2200 instrument and pore size surface area analyzer (Anton Paar QuantaTec Inc., Taipei, Taiwan). The Brunauer–Emmett–Teller (BET) specific surface area (*S_BET_* (m^2^/g)) was calculated from the linear part of the BET plot.

##### Thermogravimetric Analysis

Samples (10–20 mg) were placed into the ceramic pans and heat from 50 °C to 800 °C with a heat rate of 10 °C/min under nitrogen atmosphere (20 mL/min). Temperature vs. percent weight loss was graphed to determine the change between the loaded and unloaded MOF.

##### Powder X-ray Diffraction (PXRD)

X-ray Diffraction patterns of the unloaded and loaded MOF were compared to confirm the presence of magnolol in the particle. The patterns were determined at 30 kV and 10 mA with monochromated Cu Kα radiation and a scan speed of 0.5–3.5 s/step and a step size of 0.03°. Two thetas were determined at 5° to 50° using D2 Phase Bruker (Bruker, Taiwan) [36].

##### Scanning Electron Microscopy (SEM)

Samples were dried under vacuum and mounted into carbon double adhesive tape. The samples were coated with platinum under argon atmosphere and reduced pressure to increase the conductivity of the sample. The analysis was done at 5000 to 100,000 magnifications with 10,000 accelerating voltage using field emission scanning electron microscopy (JSM-7600F) (JEOL, Hsinchu, Taiwan). Micropictographs were obtained for every sample.

##### Particle Size Determination

Samples were diluted with ultrapure water with a refractive index of 1.33 at 25 °C and 78.304 dielectric constant to make 1000 ppm concentration and sonicated for 10 min at 40 kHz to facilitate distribution of the particles. Particle size was determined using the dynamic light scattering method (Nanoplus 1, Micromeritics Particulate Systems) (GatScientific Sdn Bhd, Selangor, Malaysia).

#### 2.3.4. In Vitro Drug Release

Mag@Uio-66(Zr) was subjected to the in vitro drug release study using sample and separate method [37]. Approximately 20 mg of MOF carrier containing magnolol was submerged into 20 mL release media (0.1 M hydrochloric acid (pH 2.0), 1.0 M phosphate buffered saline pH 7.4 and 6.8) confined in sealed 20 mL capacity glass vials maintained at 37 ± 0.5 °C with a constant stirring at a rate of around 75 rpm. An aliquot amount of 100 µL was taken during 0.5, 1, 2, 3, and 4 h time interval. A fresh amount of release media was used to compensate for the amount of release media taken every time points. The aliquot was assayed to determine the amount of magnolol released throughout the experiment using the HPLC method described above. The release percent of magnolol was calculated according to the following equation: percent magnolol release = {(actual amount of magnolol/loaded amount of magnolol) × 100} [35].

The cumulative released amount of magnolol from the MOF was utilized to predict and correlate the behavior of the in vitro release. The experimental data were fitted to five predictable models: zero-order, first-order, Higuchi, Korsmeyer–Peppas, and Hixson–Crowell models [38]. Data fitting was performed by linear regression using Microsoft Excel. The correlation coefficient (r^2^) was utilized in the criterion for selecting the best model that describes the release profile in the three media. The value of r^2^ closes to 1 signifies the best correlation.

#### 2.3.5. Acute Oral Toxicity

Animal toxicity test was done in accordance with the Organization for Economic Cooperation and Development guidelines for testing chemical compounds using the up and down method (OECD procedure 425). Prior to dosing, 2 mL of blood was extracted through tail clipping and was submitted for alanine aminotransferase (ALT) and creatinine level determinations for signs of liver and kidney damages, respectively. A dose of 2000 mg kg^−1^ was administered to a single female SD rat through oral gavage and was observed for 24 h for signs of toxicity and death. In cases where the first animal survived, an additional four rats were subjected to the same dose. A total of 5 test animals were observed in a 14 days period for signs of toxicity and death. An additional rat was administered with the solvent only to compare the variation in the animal species. Test animals that survived in the 14-day observation were euthanized in a carbon dioxide chamber followed by the harvesting of blood, liver and kidney of all test animals. The blood was once again submitted for ALT and creatinine level determination. The organs were submitted for tissue mounting and histopathological evaluation [39].

#### 2.3.6. Oral Bioavailability and Tissue Distribution

The determination of the bioavailability of magnolol and its tissue distribution to the brain, liver, and kidney was done according to the method of Ding et al. (2018), Higashi (2015), Lin et al. (2011), and Kotani et al. (2005) [13,40,41,42].

##### Collection of Serum Samples

For the pharmacokinetic study, male SD rats were randomly divided into 4 groups (*n* = 3). In particular, 2 groups were given magnolol at a dose of 100 mg kg^−1^ via oral and intraperitoneal routes; another set of two groups was given the same dose of mag@Uio-66(Zr). Blood samples (approximately 200 to 250 µL) were collected at predetermined time points (15, 30, 60, 120, 180, 240, 480, and 720 min) and allowed to clot and centrifuged at 4000 rpm at 4 °C for 5 min to obtain the serum. The obtained sera were treated with equal amount of acetonitrile and then centrifuged again to obtain the protein-free sera.

##### Collection of Tissue Samples

For the tissue distribution study, male SD rats were divided into two groups (*n* = 3) and were given either magnolol or mag@Uio-66(Zr) orally administered at a dose of 50 mg kg^−^^1^. After one hour of administration, the animals were euthanized in a carbon dioxide chamber and their organs (brain, liver, and kidney) were collected. The organs were weighed and homogenized in 10 mL of acetonitrile, and centrifuged at 10,000 rpm for 10 min. The supernatant was dried under reduced pressure.

##### Magnolol Quantification

Samples were reconstituted with acetonitrile prior to HPLC analysis. The samples were injected into the HPLC (*n* = 3). The quantity of magnolol was computed using a standard calibration curve.

#### 2.3.7. Statistical Analysis

All experiments were conducted triplicates. Statistical analysis of significance was performed using the SPSS 19.0 statistical software (SPSS Inc., Chicago, IL, USA). Differences within the group were evaluated using paired sample t-test, while differences between various groups were evaluated using one-way analysis of variance (ANOVA), and *p*-value < 0.05 indicated statistical significance. Post hoc analysis was done using Tukey HSD.

## 3. Results

### 3.1. Magnolol Impregnation and Quantification

**Figure 2:**
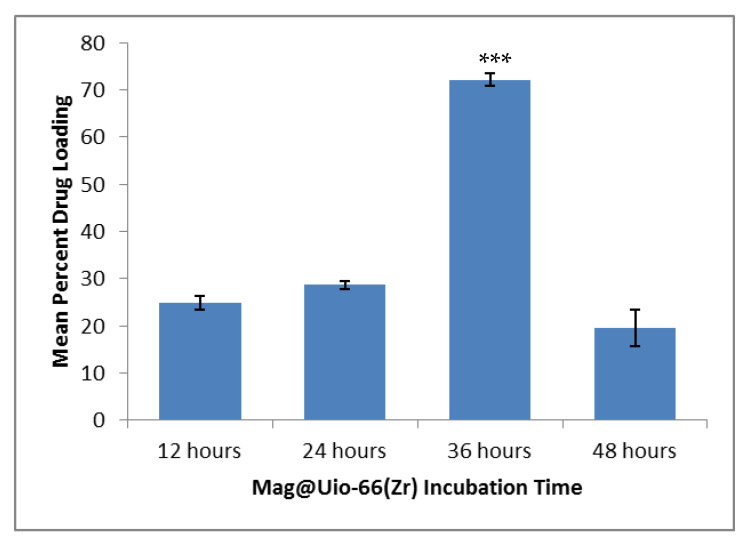
Mean drug loading efficiency of magnolol at four different time points. *n* = 3. Data presented as mean ± S.E.M.; *** significant difference at 0.05 *p*-value.

The crystalline yellow magnolol (**1**) was impregnated to Uio-66(Zr) (**2**) and the residual magnolol was quantified using HPLC. Upon complete impregnation of magnolol, a color change was observed from white [pure Uio-66(Zr)] to slightly yellow [mag@Uio-66(Zr)] powder. This color change is due to the presence of excess magnolol. The mean drug loading efficiencies at four-time points depicted significant differences (*p* < 0.001). In particular, the mean drug loading efficiency at 36 h time point is significantly greater compared to other three time points (*p* < 0.001), as shown in Figure 2. A time-dependent drug loading efficiency of Uio-66(Zr) was observed until the 36th hour (24.86 ± 2.43% < 28.64 ± 1.59% < 72.16 ± 2.15%). However, efficiency drastically decreased after 48 h (19.51 ± 6.85%).

### 3.2. Characterization of mag@Uio-66(Zr)

#### 3.2.1. Nitrogen Sorption Isotherms

**Figure 3:**
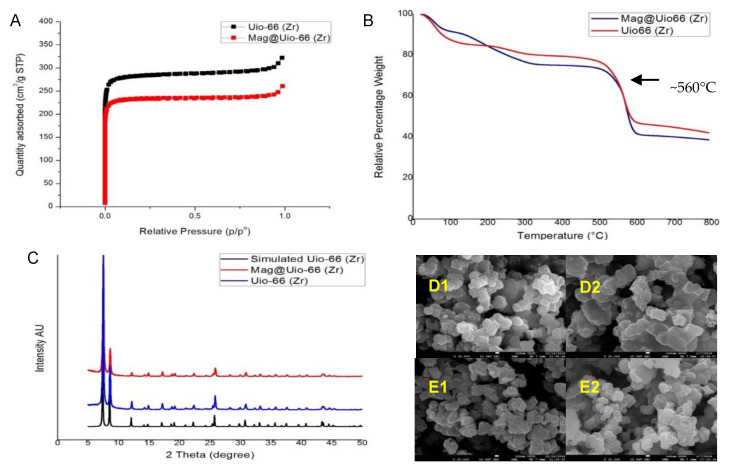
Characterization of pure Uio-66(Zr) and mag@Uio-66(Zr) after 36 h. (**A**) nitrogen adsorption–desorption plot, (**B**) thermogram plot, (**C**) XRD pattern, (**D1**–**2**) micropictograph of pristine Uio-66(Zr) at 30,000×, and (**E1**–**2**) micropictograph of mag@Uio-66(Zr) at 30,000×.

Nitrogen adsorption-desorption isotherms were obtained and analyzed to determine the Brunauer–Emmett–Teller (BET) surface areas of the pure Uio-66(Zr) and mag@Uio-66(Zr) at the 36th hour incubation time. This was done to check if magnolol was successfully impregnated to Uio-66(Zr) as determined by a decrease in surface area in the MOF. Thus, a decrease in BET surface area, pore volume and pore diameter of the pure Uio-66(Zr) upon incorporation of magnolol were observed. The following changes in pore and surface properties were noted: BET surface area (from 1156.0643 m^2^/g to 951.9567 m^2^/g), pore volume (from 0.473321 cm^3^/g to 0.380273 cm^3^/g), and pore diameter (from 16.37 Å to 15.97 Å). The reduction in parameters after drug incorporation is attributed on the impregnation of magnolol in the Uio-66(Zr) framework. A sharp increase in the nitrogen adsorption at low pressure was noted, indicating the void space in the pristine framework. Overlapping of the adsorption–desorption isotherms in Uio-66(Zr) at low pressure indicates a microporous structure, as shown in Figure 3A [35]. 

#### 3.2.2. Thermogravimetric Analysis (TGA)

Thermal degradation of Uio-66(Zr) and mag@Uio-66(Zr) was studied through TGA to check the incorporation of magnolol in the MOF framework. Pure Uio-66(Zr) was stable up to 560 °C as shown in Figure 3B, while two thermal degradations were noted in the mag@Uio-66(Zr) thermogram. The first was attributed to the impregnated magnolol at 350–400 °C, while the second thermal degradation at ~560 °C was due to Uio-66(Zr). Shifts in the thermogram curve of the loaded and unloaded Uio-66(Zr) exhibit differences in thermal property which may be corroborated to magnolol impregnation in the MOF structure [43,44,45]. In addition, based on the relative percentage weight of mag@Uio-66(Zr), as seen in the thermogram curve in Figure 3B, there is a lower residual mass due to higher organic material (magnolol and terephthalate) lost during the course of analysis.

#### 3.2.3. Powder X-ray Diffraction (PXRD)

The crystalline integrity of Uio-66(Zr) conforming to the simulated crystalline structure and the integrity after incorporation of Uio-66(Zr) with magnolol was determined using PXRD, as shown in Figure 3C. The PXRD spectrum for the synthesized Uio-66(Zr) was identical to that of the simulated spectral patterns obtained from validated single crystal structure of Uio-66(Zr), indicating successful formation, crystallinity and phase homogeneity, as displayed in Figure 3C. The structural integrity of Uio-66(Zr) remained even after impregnation with magnolol at 36 h indicated by no change in PXRD spectral patterns between unloaded MOFs and loaded MOFs. These findings indicate the stability of mag@Uio-66(Zr) up to 36 h. Additionally, the level and intensity of the peaks were significantly reduced after exposure to magnolol. These changes with the PXRD spectral patterns were attributed to the incorporation of magnolol within the Uio-66(Zr) structure [35]. Furthermore, the absence of Bragg peaks corresponding to free magnolol rules out the presence of free recrystallized magnolol outside of the pores of the material [46]. 

#### 3.2.4. Scanning Electron Microscopy (SEM)

SEM was done to determine the microcrystallinity and textural shapes of pure Uio-66(Zr) and mag@Uio-66(Zr). Figure 3D,E shows the nature of microcrystallinity and relatively similar textural shape of the unloaded and loaded Uio-66(Zr). Uio-66(Zr) and corresponding loaded one display a quasi-spherical or nearly spherical shape. 

#### 3.2.5. Particle Size Determination

Particle sizes of the Uio-66(Zr) and mag@Uio-66(Zr) were determined. Significant increase in the particle size of Uio-66(Zr) after loading was also noted with sizes of 338.90 nm ± 18.42 nm and 500.80 ± 16.63 nm, respectively (*p* = 0.012). The increase in the particle size could be due to the loading of magnolol.

### 3.3. In Vitro Drug Release

**Figure 4:**
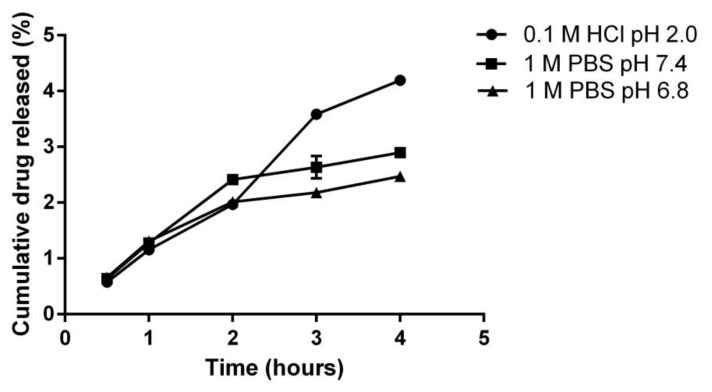
Cumulative drug release of magnolol from mag@Uio-66(Zr) in three media. HCl—hydrochloric acid; PBS—phosphate buffered saline at pH 7.4 and 6.8.

In vitro drug release of the mag@Uio-66(Zr) prepared after 36 h was carried out at 1.0 M phosphate buffered saline (PBS) at pH 7.4 (simulated blood pH) and pH 6.8 (simulated intestinal pH), and 0.1 M hydrochloric acid pH 2.0 (simulated gastric pH), as shown in Figure 4. The drug release experiment was carried out for four hours. After four hours of drug release, the cumulative percentage of magnolol released were 2.47 ± 0.37% (PBS pH 6.8), 2.89 ± 0.09% (PBS pH 7.4), and 4.19 ± 0.03% (0.1M HCl pH 2). With these cumulative values of magnolol released, it was deduced that there was a very slow release of magnolol in the simulated release media. The data were plotted to determine the release kinetic profile at zero order kinetics, first order kinetics, Higuchi model, Korsmeyer–Peppas model and Hixson Crowell model (refer to Table S1 for regression factors (r^2^) of all five models).

### 3.4. Acute Oral Toxicity

**Figure 5:**
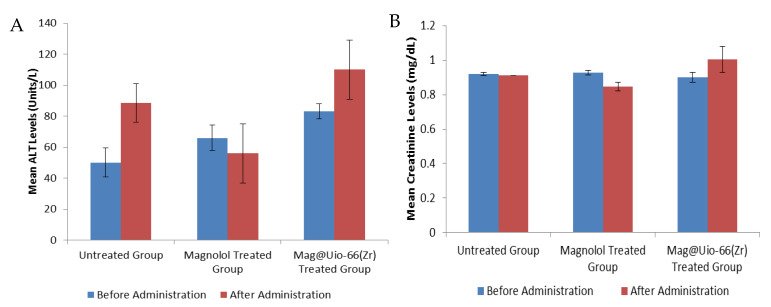
Serological parameter for acute oral toxicity. (**A**) mean ALT levels (units/L) for magnolol and mag@Uio-66(Zr) treated group, and (**B**) mean creatinine (mg/dL) levels for magnolol and mag@Uio-66(Zr) treated group. Blood extraction was done before and after administration of test compounds. *n* = 5. Data presented as mean ± S.E.M.

Acute oral toxicity was performed to check the preliminary toxicity of magnolol and mag@Uio-66(Zr) based on the Organization for Economic Cooperation and Development guidelines for testing chemical compounds using the up-and-down method (OECD procedure 425). All test animals survived the 2000 mg kg^−1^ limit dose after oral administration of both magnolol and mag@Uio-66(Zr). Therefore, the median lethal dose (LD_50_) for both magnolol and mag@Uio-66(Zr) were assumed to be greater than 2000 mg kg^−1^. To validate the result, serum (alanine transaminase) ALT and creatinine levels for magnolol- and mag@Uio-66(Zr)-treated groups were taken before and after administration of the test sample, as displayed in Figure 5. Using paired t-test analysis, the mean serum ALT level for both magnolol and mag@Uio-66(Zr) groups displayed no significant difference (*p* = 0.276 and *p* = 0.055, respectively). Interestingly, the magnolol-treated group showed a significantly lowered serum creatinine levels after oral administration (*p* = 0.440) while no significant difference in the mean serum creatinine levels for mag@Uio-66(Zr)-treated group (*p* = 0.446) was observed. The control test animal, receiving only the vehicle, exhibited no significant differences for both serum ALT and creatinine levels (*p* = 0.330 and *p* = 0.500, respectively). Interestingly, the toxicity evaluation of mag@Uio-66(Zr) had produced neither deaths nor signs of toxicity.

**Figure 6:**
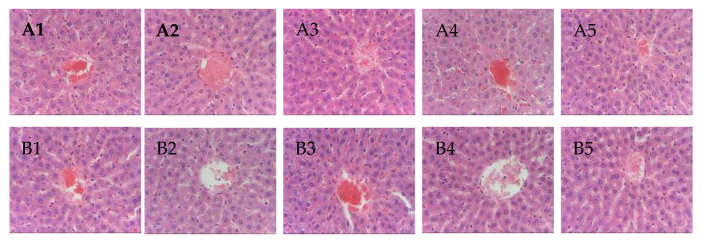
Micropictograph of the liver tissue sections for (**A**) magnolol treated group (from left to right, rat 1 to 5), and (**B**) mag@Uio-66(Zr)treated group (from left to right, rat 1 to 5).

**Figure 7:**
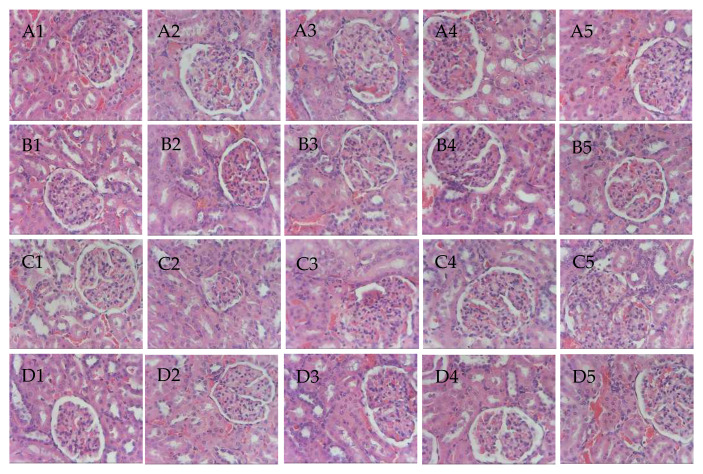
Micropictograph of the kidney tissue sections for(**A**) right kidney (magnolol treated group), (**B**) left kidney (magnolol treated group), (**C**) right kidney (mag@Uio-66(Zr) treated group), and (**D**) left kidney (mag@Uio-66(Zr) treated group) (from left to right, rat 1 to 5).

Gross necropsy revealed no signs of macroscopic damage on both livers and kidneys of the test animals. Histopathological evaluation of the liver and kidney tissue slides was done to check microscopic damages. Regional hepatocytes with the limiting plates were seen intact for all sections of the liver sample, as shown in Figure 6. Observed mild hepatocyte atrophy was only related to the general nutrition and unrelated to the test compound. Granularity and swelling of the hepatocyte cytoplasm were within limits. For all sections of kidneys shown in Figure 7, no pathological damages were observed on the glomeruli and proximal tubular epithelia. Occasional cast formations on some tubules were observed but not of significant magnitude.

Samples were stained with hematoxylin and eosin dyes. Micropictograph was taken at the periacinar hepatocyte using 40X objective in an OlympusCH2 microscope fitted with a 5MP camera.

Samples were stained with hematoxylin and eosin dyes. Micropictograph was taken at the glomeruli and proximal tubule using 40× objective in OlympusCH2 microscope fitted with a 5MP camera.

### 3.5. Oral Bioavailability and Tissue Distribution

#### 3.5.1. Oral Bioavailability

**Figure 8:**
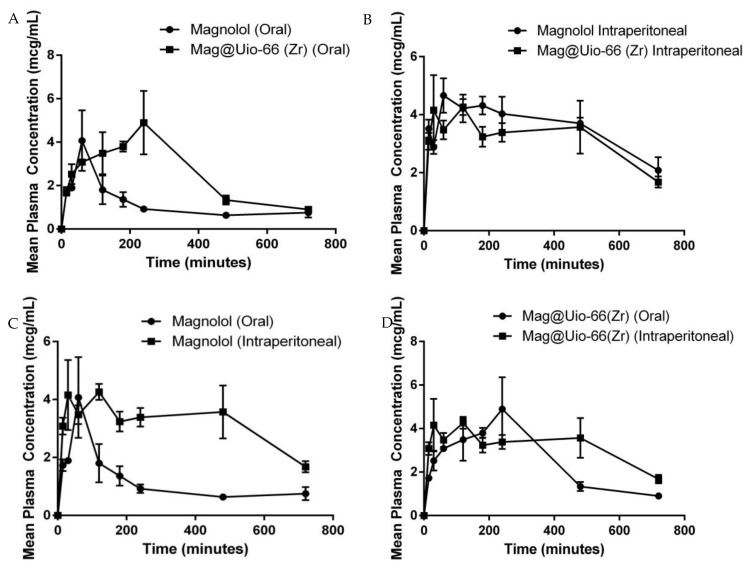
Area under the curve (AUC_0–720_) graph of (**A**) magnolol (oral) and mag@Uio-66(Zr) (oral), (**B**) magnolol (intraperitoneal) and mag@Uio-66(Zr) (intraperitoneal), (**C**) magnolol (oral) and magnolol (intraperitoneal), and (**D**) mag@Uio-66(Zr) (oral) and mag@Uio-66(Zr) (intraperitoneal). *n* = 3. Data plotted as mean ± S.E.M. in time (minutes) and mean plasma concentration of magnolol (µg/mL).

Oral bioavailability of magnolol was determined by administering pure magnolol and mag@Uio-66(Zr). The pharmacokinetic parameters of magnolol and mag@Uio-66(Zr) are shown in Table 1. Figure 8 shows the mean plasma concentration–time curve of magnolol in rats following either oral or intraperitoneal administration of 100 mg kg^−1^ of magnolol and mag@Uio-66(Zr). To have insights on the plasma concentration of magnolol present in a certain amount of time, and to show systemic exposure to magnolol, the area under the curve (AUC) was determined [47]. The area under the curve from time 0 to 720 min (AUC_0–720_) of mag@Uio-66(Zr) is significantly higher than the AUC_0–720_ of magnolol-treated group (*p* = 0.010) at post oral administration, as shown in Figure 8A. Meanwhile, the AUC_0–720_ between magnolol and mag@Uio-66(Zr) after intraperitoneal route administration depicted no significant difference (*p* = 0.412), as displayed in Figure 8B. Comparing AUC_0–720_ between oral and intraperitoneal administration of pure magnolol showed a significant difference (*p* < 0.001) wherein the intraperitoneal route of administration possesses greater AUC_0–720_, as shown in Figure 8C. Interestingly, the AUC_0–720_ between the oral and intraperitoneal route of mag@Uio-66(Zr) showed no significant difference (*p* = 0.182), as shown in Figure 8D. This indicates the promising use of Uio-66(Zr) as drug carrier for magnolol as indicated by enhancement in AUC_0–720_ which is also considered comparable to the intraperitoneal route.

#### 3.5.2. Tissue Distribution

Tissue distribution study was done to check the presence of unconjugated magnolol after oral administration of either pure magnolol or mag@Uio-66(Zr) in rats. One-hour post-oral administration of 50 mg kg^−1^ of magnolol and mag@Uio-66(Zr), principal organs were obtained. No significant differences were observed for the mean magnolol concentration in the brain, liver, and kidneys of all test animals after one hour with p-values of 0.361, 0.299, and 0.051, respectively, as shown in Table 2.

## 4. Discussion

**Figure 9:**
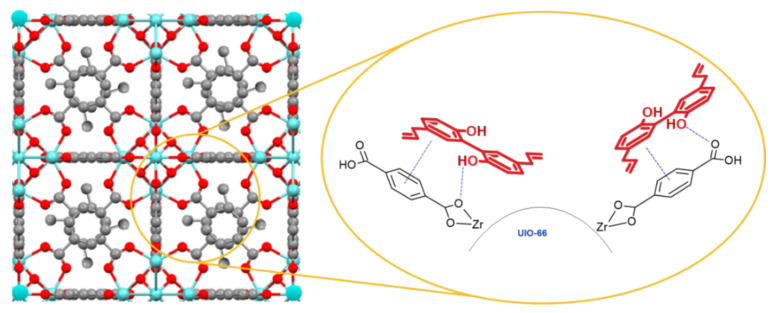
Predicted binding mechanism of magnolol to Uio-66(Zr) [51] showing π–π interaction and hydrogen bonding.

This is the first study utilizing Zr-based metal organic framework as a drug carrier for magnolol. Uio-66(Zr) (**2**) exhibited time-dependent loading of magnolol up to 36 h due to longer exposure of the MOF to magnolol (**1**), accounting for 72.16% *w*/*w* (~0.721mg of magnolol present per milligram of Uio-66(Zr)). However, a drastic decrease in the loading efficiency was observed, suggesting that longer exposure to the nucleophilic hydroxyl group of magnolol affects the integrity of the MOF structure [48]. The high entrapment efficiency of magnolol in the Uio-66(Zr) is mainly attributed to its high BET surface area, pore volume and pore diameter (1156.0643 m^2^/g, 0.473321 cm^3^/g, and 16.377 Å, respectively). Magnolol was effectively incorporated into the Uio-66(Zr) framework as the magnolol diameter is 12.99 Å (as calculated, via semi-empirical optimization, using ACD/ChemSketch) compared with the previously obtained pore diameter of Uio-66(Zr) from the nitrogen absorption–desorption analysis (16.377 Å), thus allowing entry of magnolol. Another key consideration for the incorporation of magnolol is the amphiphilic nature of the internal environment within MOF allowing the incorporation of both hydrophilic and hydrophobic drugs [49]. Possible host–guest interactions include hydrogen bonding and π-π interactions, as illustrated in Figure 9 [50]. Two hydrogen binding sites are present in the magnolol structure located at the two terminal hydroxyl group attached to the benzene ring. Additionally, the π-π interactions between magnolol and the organic ligands enable the higher loading efficiency of magnolol in the Uio-66(Zr).

Similar findings were noted by previous researches using Uio-66(Zr) as its drug carrier (aspirin [46], ibuprofen [46,52], caffeine [53], and doxorubicin [54]). Additionally, the use of Uio-66(Zr) in entrapping doxorubicin [54], and alendronate [55] produces a 100% drug loading (~1mg of drug per milligram of Uio-66(Zr)). These findings support that magnolol being a smaller molecule compared to ibuprofen, caffeine, doxorubicin, and alendronate can be easily incorporated into the Uio-66(Zr) framework. This work presents better drug loading capacity of magnolol into the MOF compared to unfunctionalized and functionalized mesoporous silica (5.5% and 12.5%, respectively) [14], binary micelle system (SOL-HS15: 4.12%, and SOL-TPGS:4.03%) [13], Pluronic micelle system (27.58%) [12], polyketal microparticles (Mag-PK3: 7.86% and Mag-PLGA: 6.16%) [56], and ultrafine fibrous mats (20–30%) [57] but lower than hydrogel nanoparticles (91.6%) [15].

**Figure 10:**
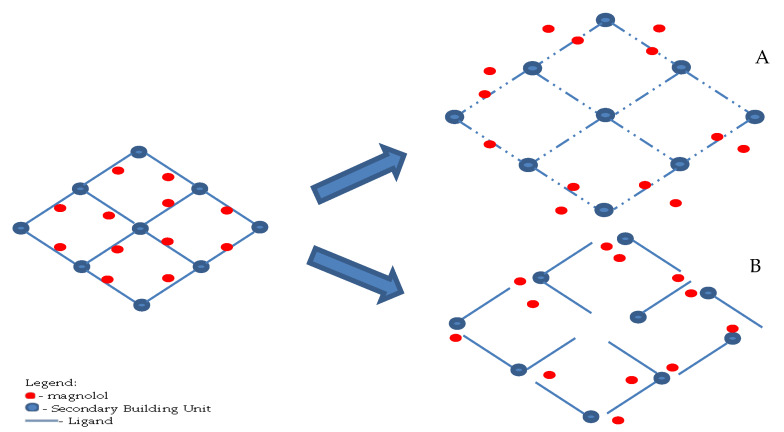
Illustration of the release of magnolol from mag@Uio-66(Zr) depicting (**A**) swelling and diffusion and (**B**) tension and breaking.

The release of magnolol in three different media resulted to 2.47% ± 0.37% (PBS pH 6.8), 2.89 ± 0.09% (PBS pH 7.4), and 4.19 ± 0.03% (0.1N HCl pH 2) after four hours. These data show that Mag@Uio-66(Zr) preparation was pH dependent. It was noted previously that Uio-66(Zr) when exposed to 0.1M HCl resulted in the protonation of the carboxylate groups present in the organic linker (terephthalic acid), suggesting that chloride ions provide charge compensation to the framework. This results to an incomplete reversible structural breakdown due to the four Zr-O bonds between each terephthalic acid [58,59]. Degradation of Uio-66(Zr) in PBS pH 7.4 and 6.8 is mainly driven by the presence of a strongly coordinating anion, phosphate ion. The phosphate ion displaces the organic linker attached to the secondary building unit leading to the breakdown of the framework and formation of zirconium phosphate salts [33,60]. Moreover, chloride ion is more abundant in the extracellular matrix compared to phosphate. In the study of Rojas and co-workers [46], ibuprofen-Uio-66(Zr) preparation showed a slow release pattern. This slow release pattern is attributed to the hydrophobicity of both host and guest molecules, resulting in slower water diffusion into the pores. This can be correlated with the relatively slow release of magnolol, since magnolol and ibuprofen are similar in structure. The data were fitted into five different mathematical models to determine the mechanism of drug release. Among these models, the Higuchi model best fits all three media with regression factor (r^2^) factors of 0.9343, 0.9389, and 0.9685 for PBS pH 7.4, pH 6.8 and 0.1M HCl pH 2, respectively. The Higuchi model describes that the release pattern of magnolol is mainly driven by diffusion-controlled mechanism (dissolution- and diffusion-controlled) [38,61,62]. To further illustrate the mechanism of the diffusion-released mechanism, the Korsmeyer–Peppas model was utilized. The kinetic constant (K) for both PBS pH 6.8, and 7.4 are 0.8442 and 0.6919, respectively, while for 0.1N HCl, it is 0.9922. Due to the nearly spherical shape of Uio-66(Zr), the spherical geometry was utilized to identify the transport mechanism. Both PBS are characterized to show an anomalous transport (*n* = 0.43–0.85) and super case II transport (*n* > 0.89) in the case of 0.1N HCl [63]. Anomalous or non-Fickian transport shown in Figure 10A, describes the mechanism of drug release that is governed by diffusion and swelling in which the rates are comparable. The time-dependent release is mainly dependent on the rearrangement of the polymeric chains and the diffusion process. In comparison with the super case II shown in Figure 10B, tension and breaking of the polymeric chain occurs as the speed of solvent diffusion is greater than the polymeric relaxation, causing an accelerated solvent penetration. This supports the effect of the ionization of the ligand at a lower pH resulting to breaking of the framework [64]. However, the composition of the release media and actual body fluids differ and greatly affect the drug release kinetics, thus, a direct comparison is not possible [46]. The cumulative amount of magnolol released did not reach more than 90%, probably due to the presence of the drug inside some micropore, covalent and π–π interactions [35].

The two degradation patterns of MOFs were breaking of metal-ligand bonding, leading to the formation of more stable complex or compound compared to the pristine MOF [65,66]. MOFs were generally formed from the Lewis acid-base (nucleophile-electrophile) interaction, thereby the presence of a more nucleophilic compound triggers the collapse of the framework as stronger nucleophile tends to bind to the electrophile, forming a more stable compound [58,67]. Proteins possess a variety of nucleophilic side chains that can react to the MOF structure, leading to a water-soluble compound [68]. Collapse of the MOF structure leads to the release of the secondary building unit (SBU) that contains the metal ion. These SBUs are characterized to have thermodynamic stability, and mechanical and architectural stability that ensures that the metal ion is strongly bound inside the molecule [69]. Release of Zr_6_O_4_(OH)_4_ happens upon the degradation of Uio-66(Zr) as this molecule serves as the SBU [58]. Lastly, zirconium has been reported to possess a very low systemic toxicity due to its poor water solubility [27,70].

Along with the AUC_0–720_ and AUC_0–__∞_, mag@Uio-66(Zr) showed a significant increase in the maximal time reaching the maximal plasma concentration (Tmax), maximal plasma concentration (Cmax), elimination half-life (T_1/2_), and absorption half-life (abs T_1/2_) compared to magnolol through the oral route (*p* < 0.001, *p* = 0.044, *p* = 0.037, and *p* < 0.001, respectively). However, only the Tmax and abs T_1/2_ of mag@Uio-66(Zr) showed a significant increase compared to magnolol through the intraperitoneal route (*p* = 0.07, and *p* = 0.042, respectively). The relative bioavailability (F_rel_) of magnolol was increased using the Uio-66(Zr) as its carrier (0.46) compared to the free magnolol (0.2). This result suggests that the use of Uio-66(Zr) markedly increased the plasma concentration of magnolol in the blood and improved the systemic performance of magnolol, specifically in 2.22-fold (AUC_0–720_), 2.33-fold (AUC_0–__∞_) and 2.09-fold (F_rel_) increases. Additionally, the AUC_0–720_ of the orally administered mag@Uio-66(Zr) is significantly comparable with the AUC of the intraperitoneally administered magnolol and mag@Uio-66(Zr) (*p* = 0.73 and *p* = 0.304, respectively). The increased AUC and F_rel_ of mag@Uio-66(Zr) is attributed to the slow release of magnolol into the media (as indicated by the longer Tmax, T_1/2_, and abs T_1/2_) thereby facilitating good solubility of magnolol and preventing saturation of the media. Another importance in the use of Uio-66(Zr) is its ability in permeating the cells through endocytosis, which enables it to deliver the drug into the blood stream [27]. This would strongly suggest that the rate limiting factor for the use of magnolol can be addressed without compromising the ease of administration. Furthermore, the use of Uio-66(Zr) prolonged the duration of the drug in the blood as indicated by a longer Tmax, T_1/2_, and abs T_1/2_. The Tmax of mag@uio-66(Zr) (3.26 h) is longer compared to other related drug delivery system of magnolol using Pluronic F127-L61 (0.75 ± 0.158) [12], SOL-HS15 micellar system (0.708 ± 0.188) [13], and SOL-TPGS micellar system (0.750 ± 0.158) [13]. Along with the data of the in vitro drug release, this would indicate that the use of Uio-66(Zr) as drug carrier can provide a sustained and controlled release of drugs.

Finally, in agreement with previously mentioned pharmacokinetic parameters, the amount of magnolol detected in the organs (brain, kidney and liver) of the orally administered mag@Uio-66(Zr) were all comparable with the free magnolol. Taking into consideration that the extraction time (60 min) was relatively close with the Tmax of magnolol (55.77 ± 4.17 min) and far from the Tmax of mag@Uio-66(Zr) (196.97 ± 17.38 min), this result would support the previous data of AUC and F_rel_ regarding the better absorption of magnolol using Uio-66(Zr).

## 5. Conclusions

The study describes the utilization of MOF as drug carrier for magnolol. Experimental outcomes suggested that MOF can be used as carrier for molecules due to their relative high surface area. The study suggests that mag@Uio-66(Zr) possesses a relatively slow release pattern but this should be confirmed with the complete release profile of magnolol. The bioavailability of magnolol was increased using Uio-66(Zr) as its drug carrier without compromising the safety of magnolol. Systemic performance was enhanced based on the increased AUC_0–720_, AUC_0–__∞_, and (F_rel_) for magnolol upon utilization of Uio-66(Zr). With the data from the in vitro drug release and the prolongation of the Tmax, this would suggest that the use of Uio-66(Zr) as drug carrier can lead to a prolonged released of magnolol. Metal organic framework serves as a new avenue for a drug delivery system that is nontoxic and effectively delivers poorly soluble drugs into the blood.

## Figures and Tables

**Table 1:** Summary of the pharmacokinetic parameters for oral and intraperitoneal route of magnolol and mag@Uio-66(Zr).

Test Compound	Parameters					
	AUC_0–720_ (µg/mL min)	AUC_0–__∞_ (µg/mL min)	Tmax (min)	Cmax (µg/mL)	T_1/2_ (min)	Abs T_1/2_ (min)
Magnolol PO	823.3 ± 139.10	903.97 ± 140.09	55.77 ± 4.17	2.57 ± 0.26	100 ± 20.40	23.26 ± 3.16
Mag@Uio-66(Zr) PO	1823 ± 167.31 *	2099.95 ± 148.48 *	196.97 ± 17.38 *	3.77 ± 0.33 *	206.21 ± 27.95 *	118.92 ± 6.22 *
Magnolol IP	2582.67 ± 150.48	4016.90 ± 535.62	64.06 ± 6.88	5.10 ± 0.65	460.88 ± 37.41	17.14 ± 3.63
Mag@Uio-66(Zr) IP	2312.67 ± 253.76	3831.72 ± 451.57	114.27 ± 7.09 **	5.65 ± 2.41	606.35 ± 114.37	33.26 ± 4.09 **

*n* = 3; Data presented as mean ± S.E.M. * significant difference between oral route at 95% confidence interval; ** significant difference between intraperitoneal route at 95% confidence interval; PO—oral route; IP—intraperitoneal route; AUC_0–720_—area under the curve from 0 to 720 min; Cmax—maximal plasma concentration; Tmax—maximal time reaching the maximal plasma concentration; T_1/2_—elimination half-life; Abs T_1/2_—absorption half-life.

**Table 2:** Summary of the mean magnolol concentration (µg/g) of magnolol and mag@Uio-66(Zr) in the brains, livers, and kidneys.

Test Compound	Organs		
	Brain (µg/g)	Liver (µg/g)	Kidneys (µg/g)
Magnolol	0.374 ± 0.022	0.79 ± 0.18	3.74 ± 0.89
Mag@Uio-66(Zr)	0.413 ± 0.034	1.07 ± 0.19	1.82 ± 0.21

*n* = 3; data presented as mean ± S.E.M (µg/g). Organ collected after 1 h of oral administration of the test compounds.

## Supplementary Materials

Summary of the drug release kinetic models for the in vitro drug release study. The following are available online at https://www.mdpi.com/1999-4923/12/5/437/s1, Table S1: Summary of Kinetic model constants for the three release media of Uio-66(Zr) at 4 h.
