# Supplementary material for: Enhanced Oral Bioavailability of the Pharmacologically Active Lignin Magnolol via Zr-Based Metal Organic Framework Impregnation
Source: Pharmaceutics. 2020 May 9;12(5):437. doi: 10.3390/pharmaceutics12050437 (PMC7285002; doi:10.3390/pharmaceutics12050437)
Supplement: Supplementary file 1 [file pharmaceutics-12-00437-s001.pdf]

pharmaceutics

Supplementary Materials: Enhanced Oral Bioavailability of the Pharmacologically Active Lignin Magnolol via Zr-Based Metal Organic Framework Impregnation

Joshua H. Santos, Mark Tristan J. Quimque, Allan Patrick G. Macabeo, Mary Jho-Anne T. Corpuz, Yun-Ming Wang, Tsai-Te Lu, Chia-Her Lin and Oliver B. Villaflores

In-Vitro Drug Release and Drug Release Kinetic Models

In-vitro drug release of the mag@Uio-66(Zr) prepared after 36 hours was carried out at 1.0 M phosphate buffered saline (PBS) at pH 7.4 (simulated blood pH) and pH 6.8 (simulated intestinal pH), and 0.1 M hydrochloric acid pH 2.0 (simulated gastric pH).

Table S1. Summary of kinetic model constants for the three release media of Uio-66(Zr) at 4 h.

Model Name	1M Phoshate Buffered Saline pH 7.4			1M Phosphate Buffered Saline pH 6.8			0.1M HCl pH 2.0		
	R^2	K	y	R^2	K	y	R^2	K	y
Zero Order	0.9623	0.7746	−0.234	0.8852	0.5881	0.4107	0.9874	1.0797	0.0239
First Order	0.9045	−0.0032	1.9984	0.887	−0.0026	1.9982	0.9874	−0.0048	1.999
Higuchi	0.9343	1.6974	0.1536	0.9389	1.5028	0.2142	0.9685	2.5371	−0.2812
Korsmeyer-peppas	0.9552	0.8442	0.0622	0.9389	0.6919	0.0501	0.9828	0.9922	0.0399
Hixson Crowell	0.92	−0.0034	1.9985	0.887	-0.0026	1.9982	0.9874	−0.0169	4.6413

R^2 – regression factor; K – Kinetic constant; y – y-intercept.
